# Nervonic acid alleviates radiation-induced early phase lung inflammation by targeting macrophages activation in mice

**DOI:** 10.3389/fimmu.2024.1405020

**Published:** 2024-12-11

**Authors:** Chenlin Wang, Yanan Wu, Chao Liu, Yang Li, Song Mi, Xiaofan Yang, Tong Liu, Yuanjing Tian, YingYing Zhang, Pingping Hu, Lili Qiao, Guodong Deng, Ning Liang, Jinyue Sun, Yan Zhang, Jiandong Zhang

**Affiliations:** ^1^ Department of Oncology, The First Affiliated Hospital of Shandong First Medical University & Shandong Provincial Qianfoshan Hospital, Shandong Lung Cancer Institute, Jinan, China; ^2^ School of Clinical Medicine, Shandong Second Medical University, Weifang, China; ^3^ Department of Oncology, Shandong First Medical University & Shandong Academy of Medical Sciences, Jinan, China; ^4^ Key Laboratory of Novel Food Resources Processing, Ministry of Agriculture and Rural Affairs/Institute of Agro-Food Science and Technology, Shandong Academy of Agricultural Sciences, Jinan, China; ^5^ School of Public Health, Shandong Second Medical University, Weifang, China; ^6^ Medical Integration and Practice Center, Cheeto College of Medicine, Shandong University, Jinan, China; ^7^ Shenzhen Research Institute, Shandong University, Shenzhen, China

**Keywords:** nuclear contamination, radiation-induced lung injury, macrophage, nervonic acid, radiation therapy

## Abstract

**Background:**

Patients receiving chest radiation therapy, or exposed to high radiation levels due to accidental nuclear leakage are at risk of radiation-induced lung injury (RILI). In innate immunity, macrophages not only exhibit certain radiation tolerance but also play an important regulatory role in the whole pathological process. Nervonic acid (NA), a long-chain unsaturated fatty acid found in nerve tissue, plays a pivotal role in maintaining normal tissue growth and repair. However, the influence of NA on RILI progression has yet to be examined.

**Aim:**

This study aimed to assess the role of macrophage subtypes in RILI and whether NA can alleviate RILI. Specifically, whether NA can alleviate RILI by targeting macrophages and reducing the levels of inflammatory mediators in mouse models was assessed.

**Methods:**

Mice RILI model was employed with 13 Gy whole thoracic radiation with or without administration of NA. Various assays were performed to evaluate lung tissue histological changes, cytokine expression, IκB-α expression and the number and proportion of macrophages.

**Results:**

Radiation can lead to the release of inflammatory mediators, thereby exacerbating RILI. The specific radiation dose and duration of exposure can lead to different dynamic changes in the number of subpopulations of lung macrophages. NA can affect the changes of macrophages after irradiation and reduce inflammatory responses to alleviate RILI.

**Conclusion:**

Macrophages play a significant role in the integrated pathological process of lung injury after irradiation which shows a dynamic change with different times. NA can protect lung tissues against the toxic effects of ionizing radiation and is a new potential functional component for targeting macrophages.

## 1 Introduction

In the recent few decades, technologies related to nuclear energy have been widely applied in many aspects. However, the use of nuclear energy also brings certain safety risks, especially nuclear accidents resulting in nuclear contamination (1). Nuclear contamination not only causes severe damage to the environment and ecosystems but also poses potential threats to human health. Potential radiation sources in the environment can lead to disorders in the respiratory system, especially the lungs. Radioactive substances release high-energy particles and electromagnetic radiation, which can penetrate human tissues and interact with cells, leading to cellular damage and death (2). In the respiratory system, lung is one of the organs most susceptible to radiation. Normal lung tissue is highly sensitive to radiation-induced damage and has little repair capacity (3). Therefore, patients under chest or whole-body radiation regimens may develop radiation pneumonitis and pulmonary fibrosis, which can reduce their survival rate and quality of life. Currently, there is no effective clinical treatment for radiation-associated lung disorders (4, 5).

Macrophages are a part of the immune system and play a crucial role in inflammatory responses and tissue repair (6). In the lungs, based on their unique anatomical structure, macrophages are classified as alveolar macrophages (AMs) and interstitial macrophages (IMs). AMs make up 55% of the lung’s immune cells and reside on the inner surface of the lungs. In contrast, IMs are mainly distributed in the walls of alveoli, interlobular septa, around bronchi, etc (6–8). They maintain homeostasis by engulfing inhaled particles and foreign pathogens, inducing the production of cytokines, and presenting antigens, which promotes the clearance of particulate antigens. Although macrophages play an important role in defending against biological invasions, an excessive number can lead to tissue damage (6, 9, 10). The pathological mechanisms and progression of RILI are complex, arising from intricate interactions between various cells and signaling pathways. Macrophages, as innate immune cells, are recruited to play a crucial role for infections after radiation injury. Changes in lung macrophages exposure radiation have been observed in the tissue injury, supporting the hypothesis that macrophage activation contributes to the pathogenesis of RILI (11).

As a very long-chain fatty acid, the name of nervonic acid (C24:1^Δ15^, 24:1 ω-9, cis-tetracos-15-enoic acid; NA) belonging to omega-9 type is attributed to that it is initially discovered in mammalian nerve tissues (12). NA can promote cell proliferation and differentiation, which is helpful for maintaining normal tissue growth and repair (12, 13). The previous study showed that NA could reduce the inflammatory response in macrophages induced by LPS, suppress the activation of key signal pathways closely related to the release of inflammatory factors in macrophages (14). The integration of metabolomics and transcriptomic results suggested that NA could exert its anti-inflammatory function significantly by regulating pro-inflammatory signaling and metabolic pathways (15). Dietary NA supplementation in mice can reduce the production of cytokines and proinflammatory chemokines, enhance capacity metabolism (12, 16). The regulation of NA on macrophage phenotype in the context of radiation-induced lung tissue changes and its impact on radiation-induced pulmonary fibrosis is not yet clear (17). Therefore, a mouse RILI model was established in this study to further investigate macrophages response in irradiated lungs. Mice in the RILI model were further administered NA to assess the changes of macrophages and inflammatory factors in lung tissues, and reveal how NA exerts its protective effect against RILI.

In this study, there is an increase in total macrophages and IMs after irradiation, with a decrease in alveolar macrophages. Radiation may cause an increase in the release of inflammatory factors which may further aggravate radiation lung injury. In contrast, NA appeared to effectively reduce RILI by regulating the function of IMs and inhibiting the activation of p-IκBα.

## 2 Materials and methods

### 2.1 Animals and study design

Female C57BL/6J wild-type (WT) mice of 6–8 weeks (18, 19), weighing 18 – 22 g, were provided by Vital River Company (Beijing, China). All mice were housed under specific-pathogen-free conditions at the animal facility of The First Affiliated Hospital of Shandong First Medical University & Shandong Provincial Qianfoshan Hospital. The experimental animals were allowed to at least 1 week of adaptive feeding, before being utilized in the experiments. All animal care and protocols were approved by the Institutional Animal Care and Use Committee of The First Affiliated Hospital of Shandong First Medical University & Shandong Provincial Qianfoshan Hospital (No. 2022-S303), in compliance with the Guide for the Care and Use of Laboratory Animals published by the US National Institutes of Health (NIH; Bethesda, MD, USA; publication No. 96-01). In order to investigate the effects of radiation on lung damage in mice, the mice were divided into two groups: non-radiation control group (CON) and single radiation group (RT) (**Table 1**, n=5 per group). To test the effect of NA on lung tissue after irradiation, nervonic acid was added to the above model of RILI. The mice were divided into 3 groups: non-radiation + corn oil (CON+OIL), radiation + corn oil (RT+OIL) and radiation + NA (RT+NA) groups (**Table 1**, n=5 per group, Corn oil and NA were given by gavage at a dose of 100mg/kg/every other day). The mice were observed daily and weighed weekly to ensure that the interventions were well tolerated.

**Table 1:** Experimental grouping and administration scheme.

Group	Number of mice (2/4/6 week)	Intervention		
		RT(13 Gy)	NA(100mg/kg/every other day)	Corn oil (100mg/kg/every other day)
CON	5/5/5	-	-	-
RT	5/5/5	+	-	-
CON+OIL	5/5/5	-	-	+
RT+OIL	5/5/5	+	-	+
RT+NA	5/5/5	+	+	-

### 2.2 Nervonic acid

Nervonic acid (NA) was provided by Shandong Academy of Agricultural Sciences, China. The chemical structure of NA was presented in **Supplementary Figure 1**. Based on the previous study, NA dissolved in corn oil was administered orally to mice at a dose of 100 mg/kg by gavage (14). The initiation of nervonic acid treatment commences on the first day following radiation. Nervonic acid were given every other day, and this regimen is maintained consistently until the end of the observation period.

### 2.3 Radiation injury

The mice were anesthetized with 3% isoflurane and irradiated with either a single dose of 0 Gy (non-radiation control and non-radiation + corn oil groups) or with 13 Gy over their whole thorax irradiation (WTI) using a RS 2000 pro Biological X-ray Irradiator (Rad Source Technologies, Buford, GA, USA). The mice were euthanized using a 30% volume per minute displacement rate of 100% CO₂ at 2, 4, 6 weeks after irradiation, and lung tissues were collected and processed for the analysis of RNA, protein expression levels, flow cytometry and histopathological examinations.

### 2.4 qRT-PCR

Total messenger RNA (mRNA) was extracted from part of freshly pulverized lung tissues using FastPure Cell/Tissue Total RNA Isolation Kit V2 (Vazyme, Nanjing, China; catalog No. RC 112- 01). The lung tissues were derived from CON group, RT group, CON+OIL group, RT+OIL group, RT+NA group. Then, HiScript III RT SuperMix for qPCR (+gDNA wiper) (Vazyme; catalog No. R323-01) was used to synthesize the complementary DNA (cDNA) according to the manufacturer’s protocol. The RT-qPCR sequences of each gene primers were as follows: β-actin, forward: 5’-GGCTGTATTCCCCTCCATCG-3’, reverse: 5’-CCAGTTGGTAACAATGCCATGT-3 ‘; tumor necrosis factor (Tnf), forward: 5’-CGGGCAGGTCTACTTTGGAG’, reverse: 5’-ACCCTGAGCCATAATCCCCT-3’; transforming growth factor beta1 (Tgfb1),forward: 5’-AGCTGCGCTTGCAGAGATTA-3’, reverse: 5’-AGCCCTGTATTCCGTCTCCT-3’; arginase1(Arg1), forward: 5’-TGTCCCTAATGACAGCTCCTT-3’, reverse: 5’-GCATCCACCCAAATGACACAT-3’; chitinase-like 3 (Chil3), forward: 5’-CTGGAATTGGTGCCCCTACAAT-3’; reverse: 5’-AGACCTCAGTGGCTCCTTCATT-3’, nitric oxide synthase 2 (Nos2), forward: 5’-CCTGCTTTGTGCGAAGTGTC-3’, reverse: 5’-CCCAAACACCAAGCTCATGC-3’. Gene expression levels were normalized to β-actin calculated by 2−ΔΔCt method (20).

### 2.5 Western blotting

Protein extraction from mice (CON+OIL group, RT+OIL group, RT+NA group) lung tissues was performed following standard protocol (21). The protein concentrations were estimated using BCA Protein Assay Kit (Solarbio, Beijing, China; catalog No. PC0020) to ensure equal loading of protein were subjected to immunoblotting. Samples were resolved on 10% sodium dodecyl sulfate (SDS) polyacrylamide gel, transferred onto a polyvinylidene difluoride (PVDF) membrane (Solarbio) and then incubated with the primary and secondary antibodies. The following primary antibodies were used: IκBα [Servicebio, Wuhan, China (CST); 1:1,000], p-IκBα (CST; 1:1,000), β-actin (Boster; 1:1,000). Finally, an enhanced chemiluminescence (ECL) reagent (Boster, catalog No. AR1173) was used to detect labeled protein bands. The density of bands was quantified by ImageJ software (version 1.62; NIH, USA).

### 2.6 Histopathological examination

Lung tissues were perfused (with PBS buffer) and then fixed overnight with 4% (w/v) paraformaldehyde in PBS (pH 7.2). Upon dehydration, lungs were embedded in paraffin, sectioned into 4-μm slices by paraffin microtome. Place the slices in a thermostat oven to dry them. Clear the paraffin from the slices in xylene. Then transfer the slices to ethanol (100%, 90%, 80%, 70%) and distilled water. Stain the slices in hematoxylin solution and rinse the slices under running tap water. Stain the samples in working eosin Y solution. Place the slices sequentially into 80%, 90%, 95% and 100% ethanol for dehydration. Clear the samples in xylene. Add a drop of neutral balsam onto the tissue, cover it with a coverslip, and allow it to set. After 24 hours, place them under a microscope for observation and photography.

### 2.7 Flow cytometry

Lung tissues with or without radiation were removed on week 2, 4, 6 after whole thorax irradiation. Lungs were manually minced with scissors, digested in a complete Roswell Park Memorial Institute (RPMI) medium containing DNase I 0.2 mg/mL, Solarbio, catalog No. D8071) and collagenase IV (1 mg/mL, Solarbio, catalog No. C8160) at 37°C in a 5% CO_2_ incubator for 1.5 h. The digested segments were grounded and filtered through a 70-mm cell strainer. The cells were collected from the interphase of an 80% and 40% Percoll gradient after spinning at 2,500 rpm for 15 min at room temperature. The CD16/32 antibody (eBioscience) was used to block the Fc receptors before surface staining. In order to detect the expression and quantity changes of macrophages, lung tissues were stained with antibodies against the following markers: Fixable Dye (AF405), CD45 (FITC), Ly-6G (PerCp-Cy5.5), CD11b (APC-Cy7), CD11c(PE-Cy7), Siglec-F(APC), F4/80(PE) (22). These antibodies were incubated at 4°C for 40 min. Cells were then resuspended in phosphate-buffered saline [PBS; 0.05% bovine serum albumin (BSA)] and fixed in 4% paraformaldehyde fixative (Solarbio). All samples were assayed by FACSAria II Cell Sorter [Becton, Dickinson, and Co. (BD) Biosciences, Franklin Lakes, NJ, USA] and data analysis was performed with FlowJo Software (Tree Star, Ashland, OR, USA).

### 2.8 Statistical analysis

Statistical analysis was performed using GraphPad Prism 8.0 (GraphPad Software, San Diego, CA, USA) and SPSS statistical software 25.0 (IBM Corp., Armonk, NY, USA). P values were determined using two-tailed Student’s t-test for comparing two groups and one way ANOVA for comparing multiple groups (23). Data were expressed as the mean ± standard deviation (SD). Statistical significance was indicated when P<0.05.

## 3 Results

### 3.1 Thorax radiation induced lung injury

**Figure 1:**
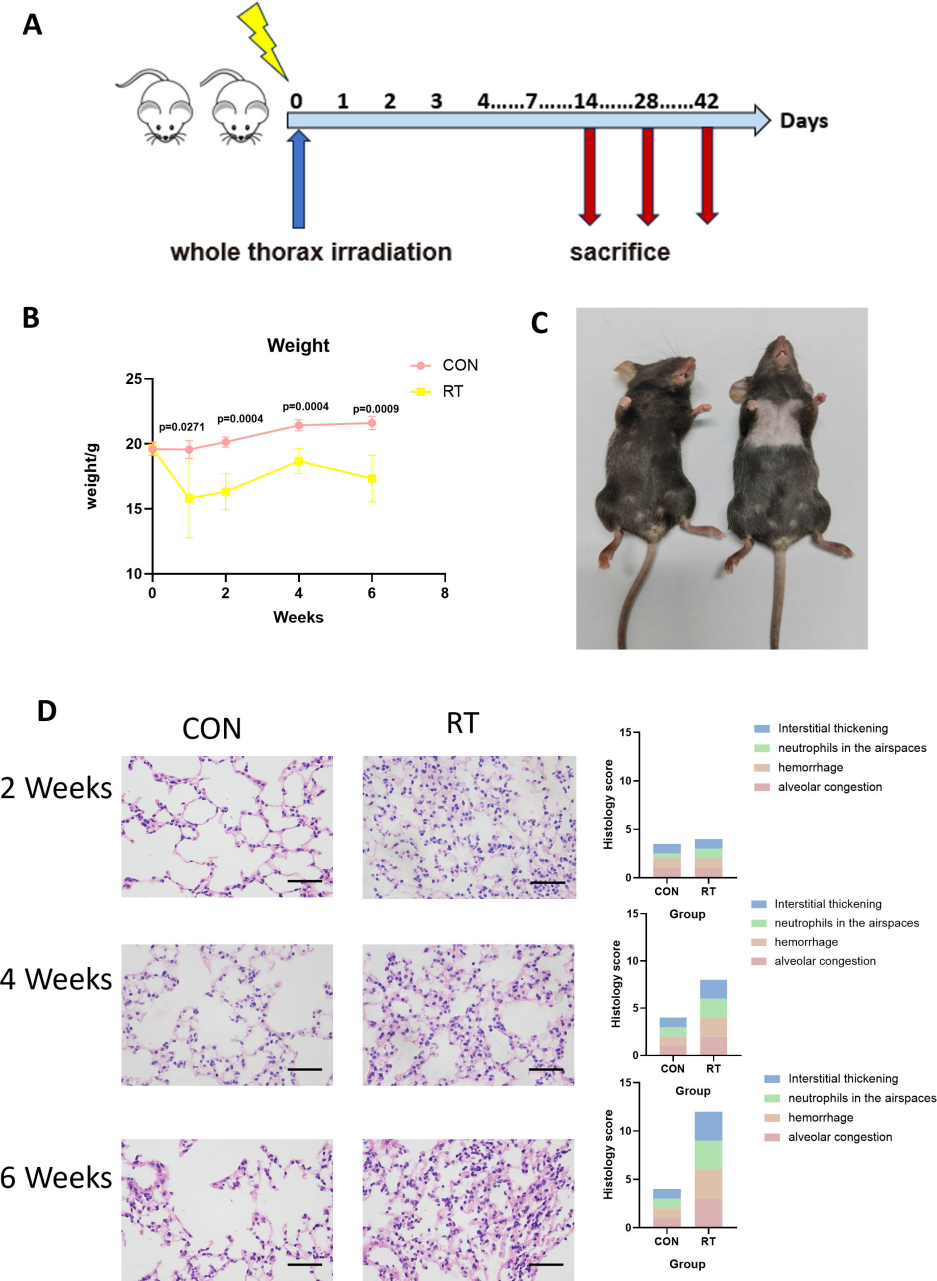
Radiation can lead to radiation-induced lung injury. **(A)** C57BL/6J wild-type mice received a single dose of whole thorax radiation (WTI, 13Gy). Lung tissues were harvested at the indicated time points for the following analysis. **(B)** Changes in body weight of non-irradiated (CON) and irradiated (RT) mice after radiation. (n=5 per group). **(C)** Changes in the hair of mice in the CON and RT groups at 6 weeks after radiation. **(D)** Lung sections of WT mice from different groups were subjected to H&E staining at 2, 4, and 6 weeks after radiation. Mice in the CON and RT groups were scored histologically. Representative images are shown (n= 5 per group). Scale bar represents 50μm. Data are presented as means ± SEM from two independent experiments. Unpaired two-tailed Student’s *t* test was used for comparison to generate *P* values in **(B)**.

To determine the effects of radiation on lung tissue, we established a mouse model of RIIL by exposing the whole lung to a dose of 13 Gy. (**Figure 1A**). The measurement of weight served as an indicator of the general health condition in mice. After 13 Gy local irradiation, changes in body weight between the radiation group and the non-radiation control group of mice were compared. At 1 week after irradiation, the weight of the mice in the radiation group decreased, and then began to grow slowly. After 6 weeks the weight of the mice decreased again. There were significant differences in body weight between the radiation and the control groups at 1 (p=0.0271), 2 (p=0.0004), 4 (p=0.0004), and 6 (p=0.0009) weeks post irradiation (**Figure 1B**). Compared with the control group, mice exhibited gradual hair loss and whitening in the area of exposure from 2 weeks. (**Figure 1C**).

Next, we analyzed H&E stained sections of lung tissue from mice. A 12-point scoring system were employed to quantitatively analyze the histopathological features of HE-stained pathological sections, assessing alveolar congestion, hemorrhage, neutrophils in airspace, and interstitial thickening (each item was scored from 0 to 3 based on severity). Compared to the control group, the radiation group showed significantly higher scores (24). At 2 weeks post irradiation, lung tissues showed congestion, and inflammatory cells in lung tissue increased. At 4 weeks, lung tissue congestion, hemorrhage, and more infiltration of inflammatory cells were observed, and to continue at 6 weeks as manifested by lung tissue destruction, interstitial thickening, and obviously severe infiltration of inflammatory cells (**Figure 1D**). Therefore, it was demonstrated that radiation led to damage in lung tissue.

### 3.2 Changes in lung macrophages after irradiation

**Figure 2:**
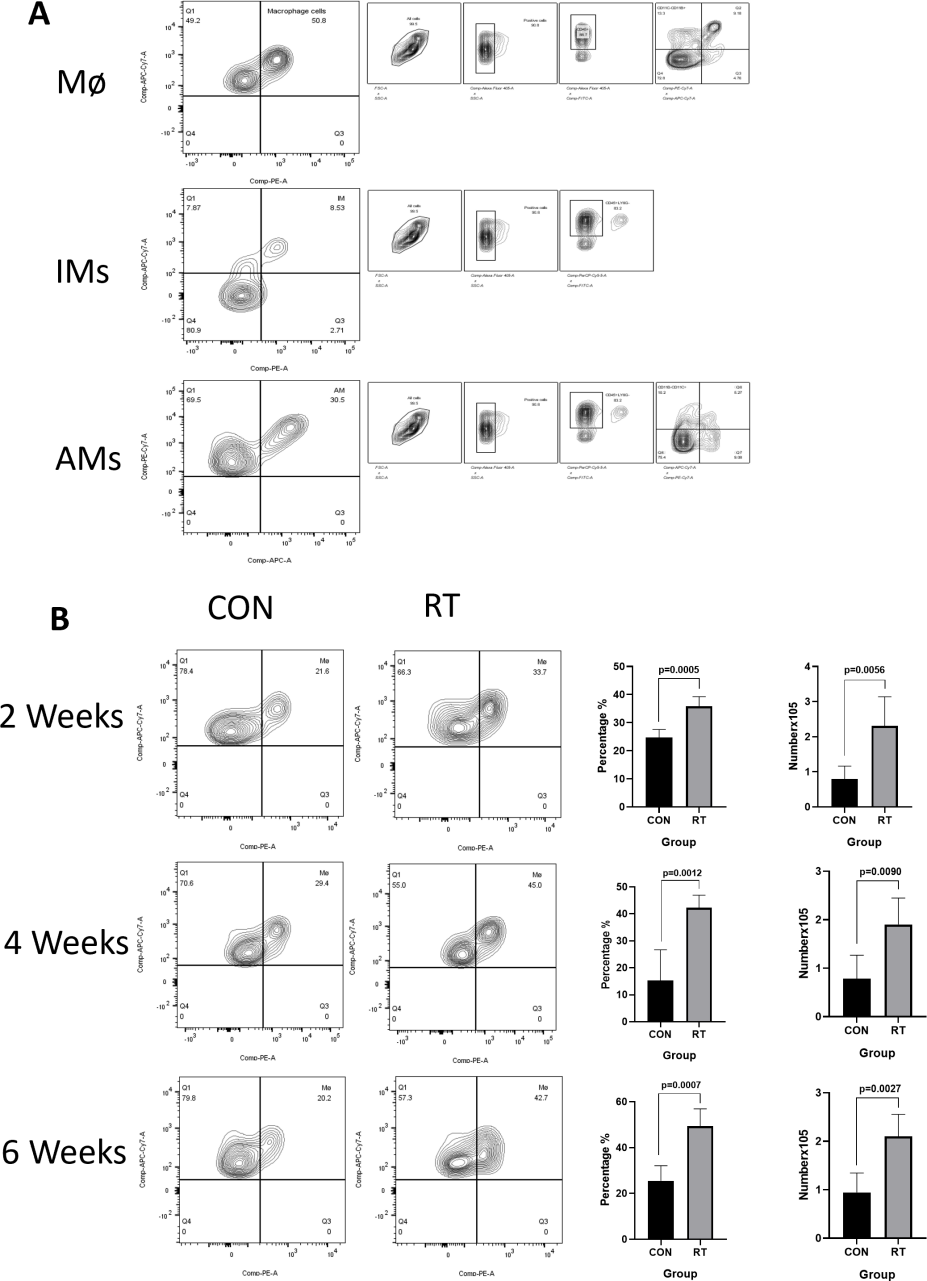
The total number of macrophages in lung tissue increased after irradiation. Whole lung cells were stained and analyzed by flow cytometry. **(A)**. Gating strategy for macrophages. Mø(CD45^+^, CD11c^-^, CD11b^+^, F4/80^+^); AMs(CD45^+^, Ly-6G^-^, CD11b^-^, CD11c^+^, Siglec-F^+^); IMs(CD45^+^, Ly-6G^-^, CD11b^+^, F4/80^+^); shown for nonirradiated sample. **(B)**. The proportion and count of total macrophages increased at weeks 2, 4, and 6 after radiation. The histogram shows the percentage and count of total macrophages. Data are presented as means ± SEM from two independent experiments. Unpaired two-tailed Student’s *t* test was used for comparison to generate *P* values in **(B)**. Mø, total macrophages; AMs, alveolar macrophages; IMs, Interstitial macrophages.

**Figure 3:**
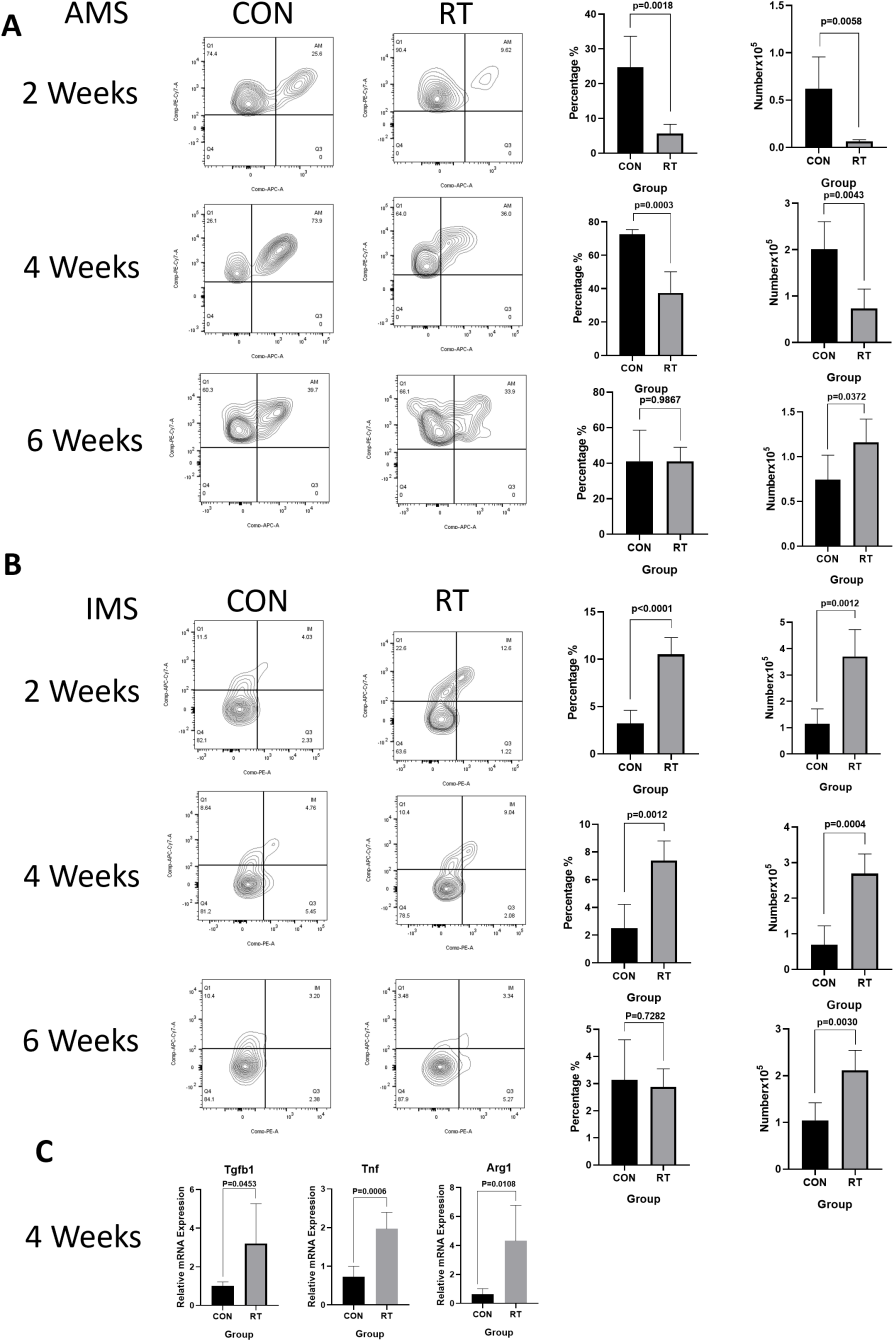
Radiation can lead to dynamic changes in macrophage subsets and increased levels of inflammatory factors in the lung tissue. Whole lung cells were stained and analyzed by flow cytometry. **(A)**. Dynamic changes of AMs after radiation. The histogram shows the percentage and count of AMs. **(B)**. Dynamic changes of IMs after radiation. The histogram shows the percentage and count of IMs. **(C)**. Analyze the level of inflammatory factors in lung tissue after radiation using qRT-PCR. The mRNA levels of Tgfb, Tnf, and Arg1 in lung tissues were upregulated after radiation. Data are presented as means ± SEM from two independent experiments. Unpaired two-tailed Student’s *t* test was used for comparison to generate *P* values in **(A, C)**. Tgfb, transforming growth factor-β; Tnf, tumor necrosis factor; Arg1, arginase 1.

To investigate the response of macrophages in RILI, the C57BL/6J mice were irradiated with a single dose of 13 or 0 Gy (control group) to the whole thorax and the lung tissues were analyzed at different time points using flow cytometry. Total macrophages were gated based on CD45^+^, CD11c^-^, CD11b^+^, F4/80^+^ staining (**Figure 2A**, the first line). The resting state of the lung is composed of AMs distributed in the alveolar cavity, whereas IMs were distributed in the interstitium. AMs were identified by a gating strategy developed by monitoring CD45^+^, Ly-6G^-^, CD11b^-^, CD11c^+^,Siglec-F^+^ (**Figure 2A**, the third line). In contrast, IMs were marked CD45^+^, Ly-6G^-^, CD11b^+^, F4/80^+^ (**Figure 2A**, the second line). Macrophages, as innate immune cells, are recruited to the site of injury where they perform their phagocytic functions. Therefore, post irradiation, macrophages are recruited to the irradiated site. The direct effects of radiation on macrophages are not yet clear (11). The results demonstrated that the total proportion and count of macrophages in the radiation group were significantly higher than those in the non-radiation group at 2, 4, 6 weeks post irradiation (**Figure 2B**). Due to the specific anatomical structure of lung tissue, the dynamic changes of AMs and IMs participate in the complex intercellular signaling transduction in the microenvironment of lung injury, and play a key role in regulating the immune response and maintaining homeostasis in the lung. To further explore the changes of AMs and IMs, further gating analysis was performed using flow cytometry. The number and proportion of AMs decreased at 2 weeks in irradiated lungs, with AMs to account for 24.68% versus 5.61% in control and radiation group, respectively (p=0.0018). The number of AMs in the control group was at 0.6224×10^5^ cells, while in the radiation group, the number was at 0.06401×10^5^ cells (p=0.0058). The number and proportion of AMs at 4 weeks in irradiated lungs still showed a downward trend, though such trend was lower than that at 2 weeks (p=0.0003 and p=0.0043). The proportion and number of AMs recovered in the lungs post irradiation after 6 weeks was measured, with proportion of AM in the control and radiation group found comparable at ca. 40% (p=0.9867). In contrast, AMs count was 0.7439×10^5^ cells in the control group and 1.164×10^5^ cells in the radiation group (p=0.0372) (**Figure 3A**). However, the number and proportion of IMs showed a different pattern with an initial increase followed by a decline. 2 weeks post irradiation, the proportion of IMs was 3.24% in the control group and 10.52% in the radiation group (p < 0.0001). The number of IMs in the control group was at 1.145×10^5^ cells and 3.704×10^5^ cells in the radiation group (p=0.0012). The number(p=0.0004)and proportion(p=0.0012)of IMs at 4 weeks post irradiation was still increased. By 6 weeks post irradiation, the proportion of IMs began to decrease. The proportion of IMs in the control group was 3.14% versus 2.88% in the radiation group (p=0.7282). The amount of IMs at 6 weeks post irradiation was lower than that at 2 and 4 weeks post irradiation. At 6 weeks post irradiation, the amount of IMs in the control group was at 1.043×10^5^ cells versus 2.166×10^5^ cells in the radiation group (p=0.0030) (**Figure 3B**).

### 3.3 Irradiation increases inflammatory factors inside the lung

At 2 and 4 weeks post irradiation, the mRNA expression of Tnf in radiation group was significantly higher (2 weeks: p=0.0289; 4 weeks: p=0.0006) than that in control group (**Figure 3C**) (**Supplementary Figure 2**). Similarly, at 2 weeks post irradiation, Arg1 mRNA increased, but there was no statistical significance(**Supplementary Figure 2**). At 4 weeks post irradiation, Tgfb1(p=0.0453) and Arg1 (p=0.0108) mRNA expression levels in the radiation group were significantly higher than those in the control group (**Figure 3C**) (**Supplementary Figure 2**). The release of other factors was likewise monitored including Chil3, and Nos2. The expression of Chil3 showed a fluctuating trend, which was down-regulated at 2 weeks and up-regulated at 4 weeks after irradiation. In contrast, no significant difference in Nos2 expression was detected (**Supplementary Figure 2**).

### 3.4 NA alleviates the lung injury post irradiation

**Figure 4:**
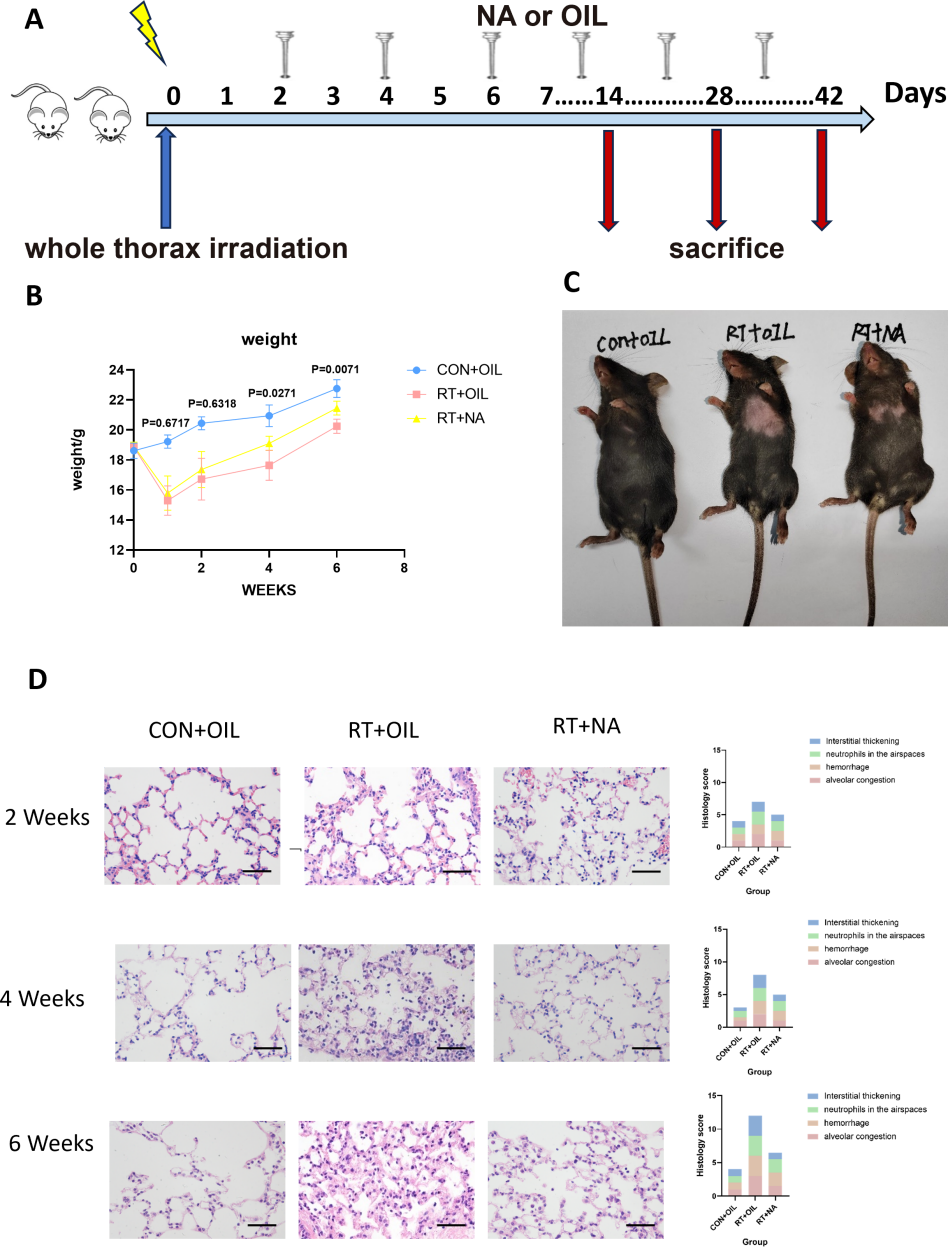
NA can alleviate radiation-induced lung injury. **(A)**. Intervention of NA on a radiation-induced lung injury model. C57BL/6J mice received a single dose of radiation and were given NA or Corn oil intragastrically every other day. Lung tissues were harvested at the indicated time points for the following analysis. **(B)**. Changes in body weight of CON+OIL, RT+OIL, and RT+NA groups of mice after radiation. (n=5 per group). **(C)** Changes in the hair of the radiation area in the chest of three groups of mice. **(D)**. Lung sections of WT mice from different groups were subjected to H&E staining at 2, 4, and 6 weeks after radiation. Mice in the CON+OIL, RT+OIL, and RT+NA groups were scored histologically. Representative images are shown (n=5per group). Scale bar represents 50μm. Data are presented as means ± SEM from two independent experiments. One-way ANOVA with Tukey’s multiple comparisons was used to generate *P* values in **(B)** (RT+OIL VS.RT+NA). NA, Nervonic acid; OIL, corn oil; RT, radiation; CON, non-radiation.

NA, a very long chain fatty acid belonging to omega-9, can promote the repair and regeneration of damaged tissue, thereby reducing inflammation caused by exogenous factors (25). To detect whether NA can alleviate RILI, the mice were randomly divided into three groups (non-radiation + corn oil, radiation + corn oil and radiation + NA groups, n=5 per group, 100mg/kg/every other day)(**Figure 4A**). At 1 week post irradiation, the body weight of the radiation group decreased, while the weight loss of the RT+NA group was less than that in the RT+OIL group. The body weight of the mice showed gradual increase, with the body weight of the radiated group found lower than that of the non-irradiated group. The difference was particularly significant at 6 weeks after irradiation (CON+OIL group vs. RT+OIL group: p<0.0001; CON+OIL group vs. RT+NA group: p=0.0045). In contrast, there was no significant difference in body weight between RT+OIL group and RT+NA group at the early stage of radiation damage (1 week: p=0.6717; 2 weeks: p=0.6318). At 4 and 6 weeks post irradiation, weight loss was significantly reduced in the RT+NA group compared to the RT+OIL group (4 weeks: p=0.0271; 6 weeks: p=0.0071) (**Figure 4B**). With the change of time after irradiation, the hair in the exposed area gradually turned white and lost, which was particularly significant at 6 weeks. In the RT+OIL group, these changes were extremely distinct compared to those of the RT+NA group. (**Figure 4C**).

To further verify the alleviating effect of NA on RILI, we performed HE staining on lung samples. As above, the slices were scored on a 12-point scale. It was found that the changes of alveolar congestion, hemorrhage, presence of neutrophils in the airspace, level of interstitial thickening, and destruction of lung tissue structure gradually aggravated with time after irradiation. Albeit, the most severe damage to the lung tissue was observed in RT+OIL group (**Figure 4D**).

### 3.5 NA affects the changes of macrophages

**Figure 5:**
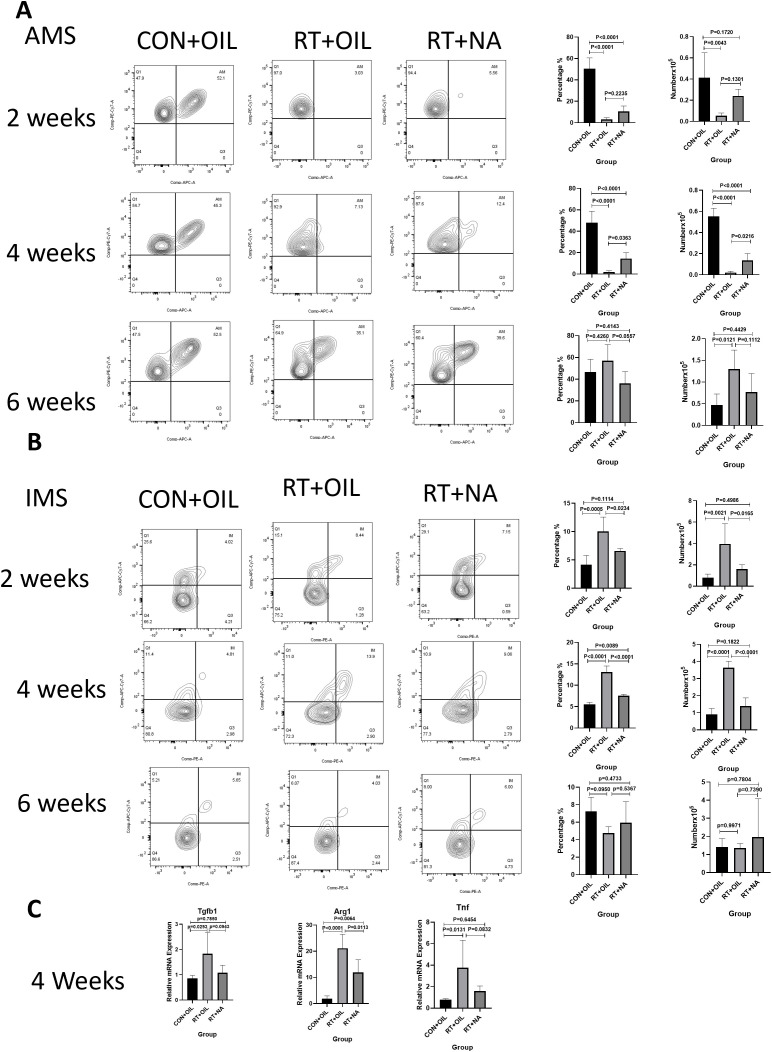
NA can modulate changes in macrophage subsets. Lung tissues were harvested at the indicated time points for the following analysis. **(A)**. NA can reduce the trend of decreased number and proportion of AMs after radiation. The histogram shows the percentage and count of AMs. **(B)**. NA can reduce the upward trend of the number and proportion of IMs after radiation. The histogram shows the percentage and count of IMs. **(C)**. NA can reduce the level of inflammation in lung tissue after radiation, as analyzed by qRT-PCR. The mRNA levels of Tgfb1, Tnf and Arg1 in lung tissue were decreased after irradiation with NA. Shown are means ± SEM (n=5 per group). One-way ANOVA with Tukey’s multiple comparisons was used to generate *P* values in **(A)** to **(C)**. NA, Nervonic acid; Tgfb, transforming growth factor-β; Tnf, tumor necrosis factor, Arg1, arginase 1.

It is well established that macrophages play a key role in the pathogenesis of RILI. In order to test whether NA could affect the response of macrophages in RILI, flow cytometric analysis was performed on lung tissue from mice at 2, 4, and 6 weeks after radiation. The gating strategy was the same as described above. At 2 weeks, the proportion and count of AMs in the RT+OIL group and RT+NA group were significantly lower than those in the CON+OIL group. Compared with RT+OIL group, the proportion and quantity of AMs in RT+NA group was found higher. This trend was more significant at 4 weeks post irradiation. The proportion of AMs in RT+OIL group was 1.98% versus 14.48% in RT+NA group (p=0.0363). The number of AMs in the RT+OIL was 0.02106×10^5^ cells and was 0.1363×10^5^ cells in the RT+NA group (p=0.0216). At 6 weeks in the irradiated lungs, the number and proportion of AMs began to rise. The AMs proportion in CON+OIL group was 46.60% versus 56.92% and 36.10% in the RT+OIL and RT+NA groups, respectively. The AMs recovery of RT+NA group was lower than that of RT+OIL group. There was no statistical significance in AMs count among all groups, but it showed an upward trend (**Figure 5A**). However, the changes of IMs were as follows: After 2 weeks of radiation, the number and proportion of IMs in the radiation group were significantly higher than those in the non-irradiated group. In the radiation group, the number and proportion of IMs in RT+OIL group were significantly higher than those in RT+NA group. The trend was more pronounced at 4 weeks. The proportion of IMs in RT+OIL group was 13.08%, and that in RT+NA group was 7.57% (p < 0.0001). The IMs count of RT+OIL group was 3.635×10^5^ cells, versus lower levels of IMs count in RT+NA group at 1.388×10^5^ cells (p < 0.0001). This trend was disrupted at 6 weeks, with both the number and proportion of IMs in the radiation group showing a decreasing trend. Although there was no statistical significance between the two groups, the number and proportion of IMs in the RT+NA group showed a lower decreasing trend than that in the RT+OIL group (**Figure 5B**). The above results indicated that NA may affect the changes in the number and proportion of macrophages, especially IMs.

### 3.6 NA reduces the level of inflammatory factors in lung tissue post irradiation

At 2 weeks post irradiation, there was no significant change in the levels of inflammatory factors in lung tissue. At 4 weeks post irradiation, Arg1 mRNA expression in RT+NA group was significantly decreased compared with that in RT+OIL group (p=0.0113). The mRNA expression levels of Tgfb1and Tnf also showed similar difference at 4 weeks, with the differences though not statistically significant (**Figure 5C**). Similarly, at 6 weeks post irradiation, mRNA expression levels of Nos2, Tgfb1, Arg1, Chil3 in RT+NA group were less than that in RT+OIL group, with a non-significant difference (**Supplementary Figure 3**).

## 4 Discussion

Due to the application and development of radiological diagnosis and treatment, alongside increase in nuclear industry applications, accidental nuclear leakage accidents, the threat of ionizing radiation to human health has become a long-term concern (26). Despite repeated screening in recent decades, the effect of radiation protection agents is still not ideal. In the C57BL/6J whole thorax irradiation model, it reported that the threshold dose for pathological changes is ranging from 9 to 20 Gy (27–33). Clinically, radiation-induced lung injury in the acute stage can result in complications such as cough, dyspnea, panting and fever, thereby limiting the dose of thoracic radiotherapy. In this research, we focused on the short-term tissue response to the acute lung injury. Therefore, a single 13 Gy dose were utilized to avoid causing serious harm to the mice. At present, there are only a few radiological protective agents in clinical application. Examples include amifostine, an FDA-approved small molecule cell protectant. However, the application of even low doses of amifostine will inevitably produce serious side effects (such as hypotension, fever, nausea and vomiting) and amifostine will be excreted rapidly by human metabolism, which greatly limits its clinical application (34). Therefore, the discovery of safe and effective radiation protective agents to treat or prevent damage caused by ionizing radiation seems warranted.

**Figure 6:**
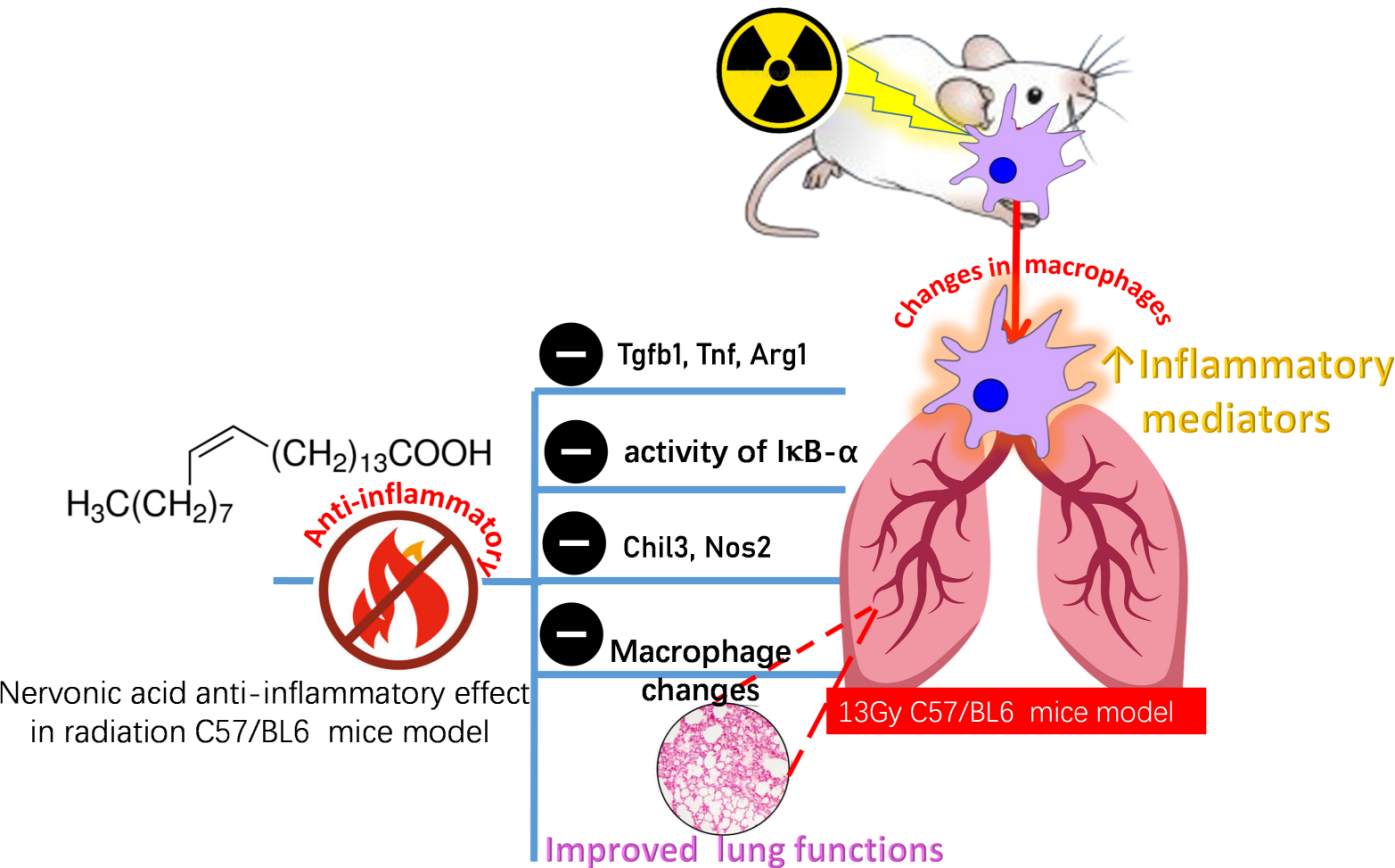
A summarizing figure for NA protective effect on lung tissues against the toxic effects of radiation. Inflammation is a key driver of radiation-induced tissue damage and an important biological event. In addition, macrophages showed a dynamic change with time after irradiation. NA can reduce the inflammatory response, inhibit the release of proinflammatory cytokines (Tgfb, Tnf, Arg1, Chil3,Nos2) reduce the phosphorylation level of the transcription factor IκBα representative of the NF-κB signaling pathway, regulate the changes of macrophages, and thus protect against radiation-induced lung injury. Tgfb, transforming growth factor-b; Tnf, tumor necrosis factor; Arg1, arginase 1; Chil3, chitinase-like 3; Nos2, nitric oxide synthase 2.

In present study, it was found that: 1. Radiation affected the changes of macrophages in the early phase after irradiation, with dynamic changes in AMs and IMs. 2. Radiation led to an increase in inflammatory factors. 3. NA alleviated RILI. 4. NA affected the changes of macrophages after irradiation and reduced the release of inflammatory factors. 5. NA is recognized as a potential agent for targeting macrophages (**Figure 6**).

Upon exposure of the human body to radiation, cells may be damaged, leading to genetic mutations, cell death, and inflammatory responses. These injuries may affect the respiratory system, especially the lungs (35). In clinical practice, it is very common for patients with thoracic tumors to have lung injury after receiving radiotherapy. Lung is one of the radiosensitive organs (36). As natural immune cells in the lung, macrophages not only have certain radiation tolerance but also play an important regulatory role in the whole pathological process. Macrophages are critical factors in inflammation and fibrosis (11, 36). Previous studies have reported that different subsets and strains of macrophages may respond differently to whole-lung radiation, which may be related to their different functions, phenotypes, and gene expression regulation (37). The specific changes in macrophages following RILI require further exploration. To understand the changes of AMs post irradiation, 13 Gy C57BL/6J mice were chosen as the animal model in this study.

Previous studies showed that after 15 days of irradiation, the number of AMs decreased to the lowest level and then increased again until week 16. In contrast, the number of IMs reached the peak on day 6 post irradiation, followed by a decrease to a sustained level until 16 weeks after irradiation, at which increase in number of IMs was detected (11, 33). The increase in IMs suggested that either IMs exerted stronger anti-radiation ability than AMs or that bone marrow mononuclear cells were replaced and supplemented more rapidly (11, 33, 38, 39). In a recent mouse post-radiation single-cell profile, single-molecule fluorescence *in situ* hybridization (smFISH) confirmed an increase in the amount of AMs and IMs in the lung 5 months post 17Gy irradiation, whereas this was not the case after 10 Gy irradiation suggestive of a dose effect in radiation response. Specific macrophage subsets were shown to be either proinflammatory or profibrotic post 17 Gy irradiation (19). Our results fall in accordance with previous reports that the number of macrophages showed an increasing trend after irradiation (6, 33, 37, 40). There was a dynamic change in the subgroup of pulmonary macrophages. After 13Gy irradiation, the AMs data showed a decrease at 2 weeks followed by a gradual increase at 6 weeks, while the IMs data showed an opposite trend. It suggested that the number of lung macrophages increased after radiation. However, the specific radiation dose and duration of exposure can lead to different dynamic changes in the number of subpopulations of lung macrophages.

Radiation can lead to the release of inflammatory factors, which can worsen radiation-induced lung injury. In the early stage post irradiation, macrophages and some foam cells are recruited inside the injured lung tissue and polarized, inducing inflammation by secreting more pro-inflammatory cytokines and promoting the development of RILI (41, 42). Macrophages can up-regulate the expression of Tnf, Nos2, IL-6, IL-1, Tgfb1, MCP, CCL2, and other factors to aggravate lung inflammation and fibrosis (21, 32, 34–37). In this study, Tnf, Tgfb1, and Arg1 were significantly upregulated 4 weeks post irradiation.

Natural active substances, due to their diversity and low toxicity, are considered of potential to reduce the damage of ionizing radiation to normal tissues (43). Omega-9, a monounsaturated fatty acid that can be obtained from several dietary sources, exhibits immunomodulatory effects and anti-inflammatory activity (44). NA was first discovered in mammalian nervous tissue and it plays an important role in human health, particularly in the health of brain (12, 45, 46). NA maintains the structure and function of biological membranes, thereby enhancing cellular viability and improving normal human activity. NA supplementation reduced the production of proinflammatory chemokines and cytokines (16, 25, 47). Wang et al. reported that NA inhibited liver inflammation *via* TNF/NF-κB signaling pathway, and these inflammatory factors included interleukin-1β (IL-1β), Bax, TNF-α, and TNF/NF-κB, IL-6, toll-like receptor 4 (TLR4) and Caspase-3 (15). A previous study showed that NA could alleviate DSS-induced colitis. *In vitro* experiments, NA could reduce LPS-induced inflammatory response of macrophages and inhibit the activation of NF-κB and MAPK signaling pathways, which are closely related to the release of inflammatory factors in macrophages. In addition, the mouse colitis model suggested that NA could decrease the levels of pro-inflammatory cytokines and inhibit NF-κB signaling pathway to improve colonic inflammation (14). These data suggested that NA is a functional dietary element with potential anti-inflammatory and immune activity and further revealed from this study regarding improvement of RILI.

In the current study, it was found that lung injury was alleviated in mice administered with NA after irradiation. Anti-inflammatory therapy mainly targets the inhibition of macrophages infiltration and the production of pro-inflammatory cytokines. To further explore the action mechanism of NA in RILI, flow cytometrically revealed for depletion of AMs in co-administered NA with radiation group compared with radiation group. In contrast, the increase in the number of IMs was lower than that in the RT+OIL group in case of NA suggestive that it target macrophages in relieving inflammation. By altering the depletion and recovery of macrophages, RILI symptoms can be alleviated. Shen-Nan Yuan et al. demonstrated that NA not only inhibited the secretion of pro-inflammatory factors IL-6, IL-1β, and TNF-α, but also promoted the release of anti-inflammatory IL-10. NA was likewise found to effectively reduce the inflammatory response in LPS-induced RAW264.7 cells (14). Macrophages release pro-fibrotic cytokines TGF-β and Arg-1, which act on fibroblasts and pericytes in the lung, promoting myofibroblast formation and extracellular matrix deposition, leading to the aggravation of pulmonary fibrosis (40, 48–50). In this study, the most significant finding was the downregulation of Arg1 in the RT+NA group inferring that NA targets macrophages to reduce Arg1 release and alleviate radiation-induced lung fibrosis in the later stages in mice.

NA can alleviate inflammation by inhibiting the activation of MAPK and NF-κB signaling pathways, classical pathways targeted in inflammation management (14). MAPK and NF-κB are two important inflammatory signaling pathways, and their activation leads to the release of inflammatory mediators, resulting in an inflammatory response. NA appeared to interact specifically with these two signaling pathways and inhibit their activation, thereby reducing the inflammatory response (14, 15, 47). Western blot analysis showed downregulation of IκBα versus an upregulation of phosphorylated IκBα in the radiation group (**Supplementary Figure 4**). These results showed that NA can alleviate RILI by targeting the function of mesenchymal macrophages, and inhibiting the activation process of p-IκBα. These findings pose NA as a functional food component that has a promising application in the prevention and treatment of RILI. Other fatty acids in the omega-9 series, such as oleic acid, have been shown to likewise exert good anti-inflammatory effects in organs such as the eyes, skin, lungs, liver, blood vessels, and intestines (44). In the future, other fatty acids in the omega-9 family are worth exploring for their role in RILI prevention.

## 5 Conclusions

Radiation triggers changes of pulmonary macrophages leading to inflammation. In this study, the dynamic change in the subsets of macrophages viz. IMS and AMS post irradiation was revealed as manifested by a decline followed by increase in case of AMS versus an opposite trend appearing in case of IMS suggestive for a differential role in lung response to radiation. In addition, NA alleviated RILI by modulating the changing of macrophages. Notably, NA appears to have the ability to inhibit the activity of IκB-α alongside other inflammatory cytokines. These action mechanisms may explain how NA potentially regulates macrophage functions, and shed light for further study of the role of NA in RILI management clinically. In general, reports on the anti-inflammatory actions of omega-9 fatty acids, other than oleic acid, are quite scarce, especially in lung disorders. Our study adds to the potential anti-inflammatory effects of omega-9 series to include NA, with these effects yet to be confirmed using randomized controlled trials to reveal solid conclusive evidence. Identification of structural motifs in NA crucial for such effects should be explored next to identify stronger analogues i.e., chain length, double bond type, and position. In addition, radiation can cause the accumulation of DNA damage and reduce the efficiency of DNA repair, and eventually lead to cell senescence or apoptosis. The potential of NA to reduce DNA damage and cell aging is worth exploring in the future.

## Data Availability

The original contributions presented in the study are included in the article/**Supplementary Material**. Further inquiries can be directed to the corresponding authors.
