# Supplementary material for: Nervonic acid alleviates radiation-induced early phase lung inflammation by targeting macrophages activation in mice
Source: Front Immunol. 2024 Dec 11;15:1405020. doi: 10.3389/fimmu.2024.1405020 (PMC11668677; doi:10.3389/fimmu.2024.1405020)
Supplement: Supplementary file 1 [file DataSheet1.docx]

Supplementary Material

Nervonic acid alleviates radiation-induced early phase lung inflammation by targeting macrophages activation in mice

**Chenlin Wang†, Yanan Wu† , Chao Liu, Yang Li, Song Mi, Xiaofan Yang, Tong Liu, Yuanjing Tian, YingYing Zhang, Pingping Hu, Lili Qiao, Guodong Deng, Ning Liang, Jinyue Sun*, Yan Zhang* and Jiandong Zhang***

*** Correspondence:**

Jinyue Sun: moon_s731@hotmail.com;

Yan Zhang: yan.zhang@sdu.edu.cn;

Jiandong Zhang: zhangjd2233@126.com

Chenlin Wang^†^, Yanan Wu^†^, These authors contributed equally to this work and share first authorship

# Supplementary Figures

## Supplementary Figure 1.


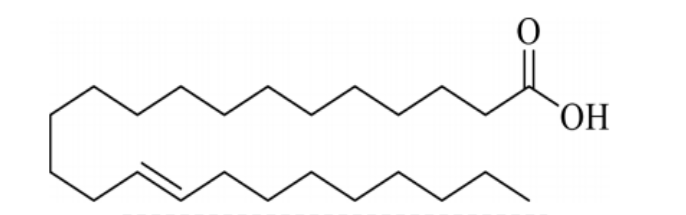


Supplementary Figure 1. The chemical structure of nervonic acid.

## Supplementary Figure 2.


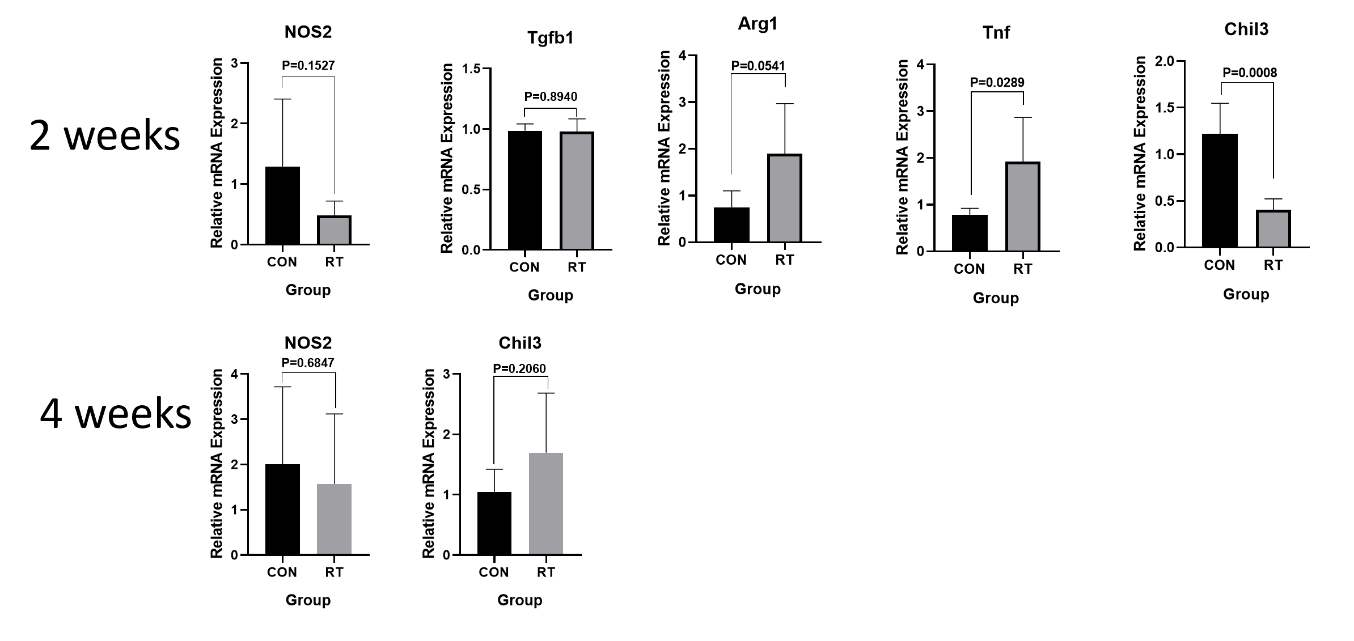


Supplementary Figure 2. Changes in the levels of inflammatory factors in lung tissue after radiation. Shown are means ± SEM(n=5 per group). Unpaired two-tailed Student’s *t* test was used for comparison to generate *P* values in Supplementary2. Nos2, nitric oxide synthase 2; Tgfb, transforming growth factor-b; Arg1, arginase 1; Tnf, tumor necrosis factor; Chil3, chitinase-like 3.

## Supplementary Figure 3.


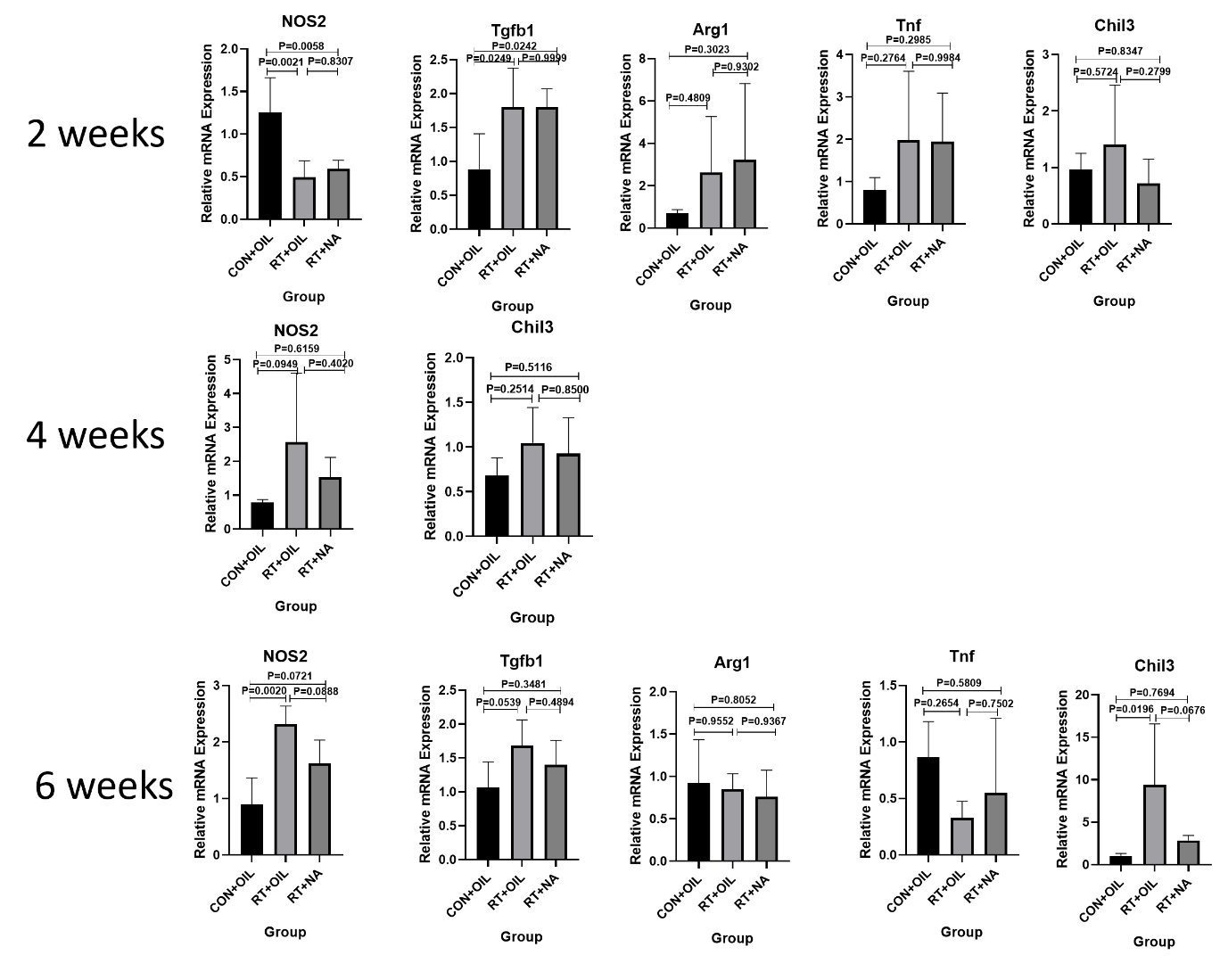


Supplementary Figure 3. The levels of inflammatory factors in lung tissue were changed after NA treatment. Shown are means ± SEM(n=4 to 5 per group). One-way ANOVA with Tukey’s multiple comparisons was used to generate *P* values in Supplementary Figure 3. Nos2, nitric oxide synthase 2; Tgfb, transforming growth factor-b; Arg1, arginase 1; Tnf, tumor necrosis factor; Chil3, chitinase-like 3.

## Supplementary Figure 4.


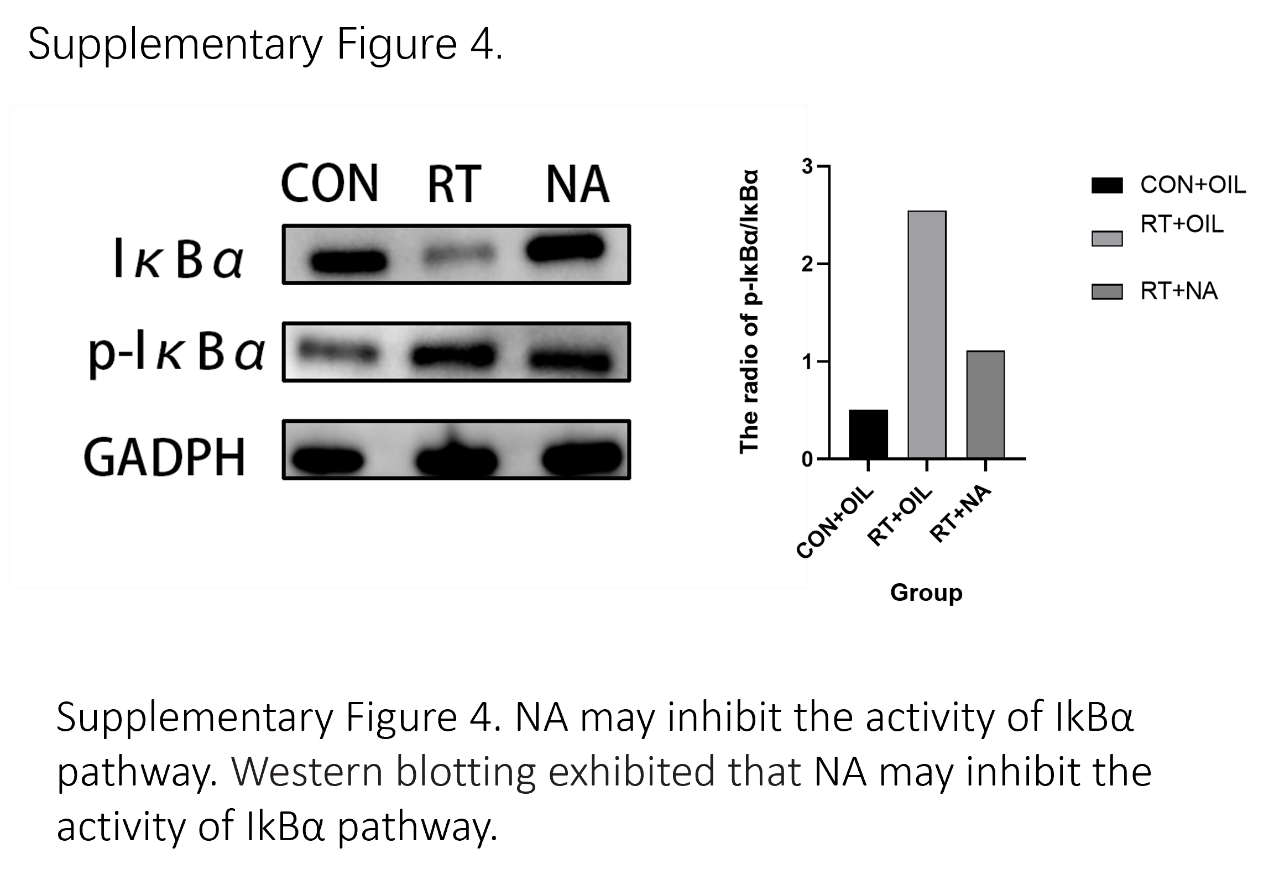


Supplementary Figure 4. NA may inhibit the activity of IkBα pathway. Western blotting exhibited that NA may inhibit the activity of IkBα pathway. The right panel is the ratio of the grayscale analysis.

**
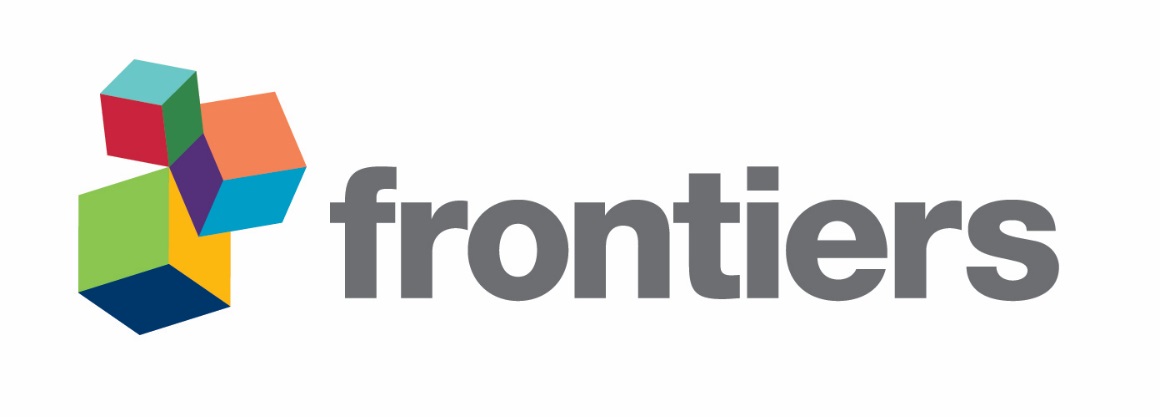
**
